# Supplementary material for: Methyljasmonate Elicitation Increases Terpenoid Indole Alkaloid Accumulation in Rhazya stricta Hairy Root Cultures
Source: Plants (Basel). 2019 Nov 22;8(12):534. doi: 10.3390/plants8120534 (PMC6963348; doi:10.3390/plants8120534)
Supplement: Supplementary file 1 [file plants-08-00534-s001.pdf]

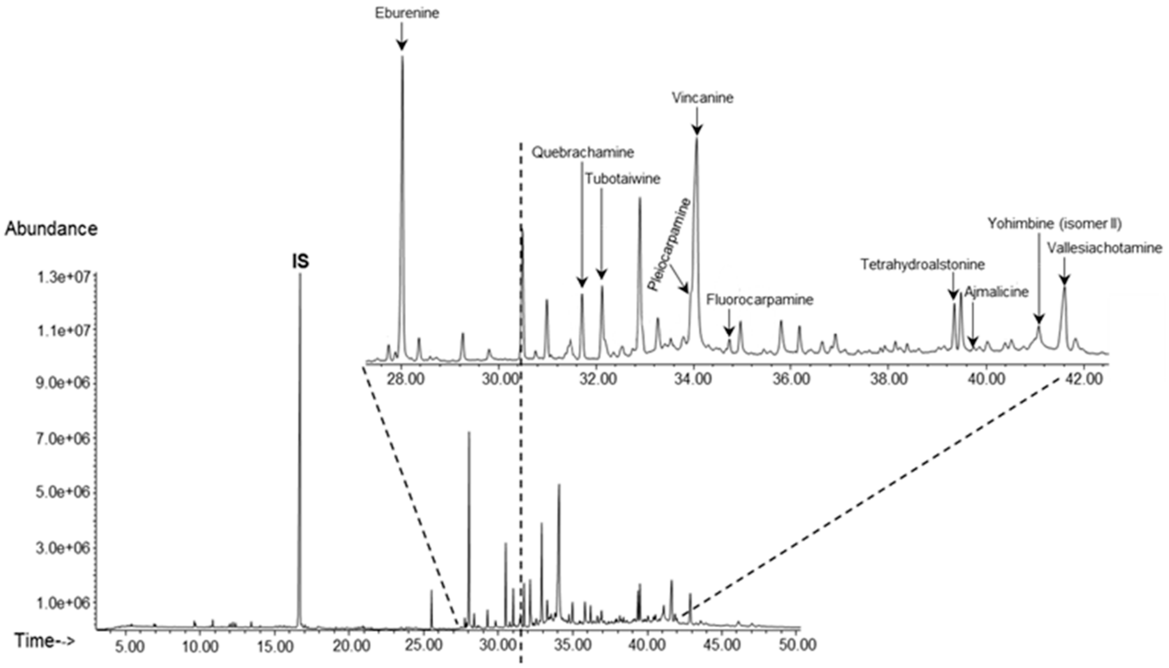

Figure 1. Total ion GC-MS analysis of *Rhazya stricta* wild type hairy root alkaloids. IS: Internal standard (2,4′-Dipyridyl).

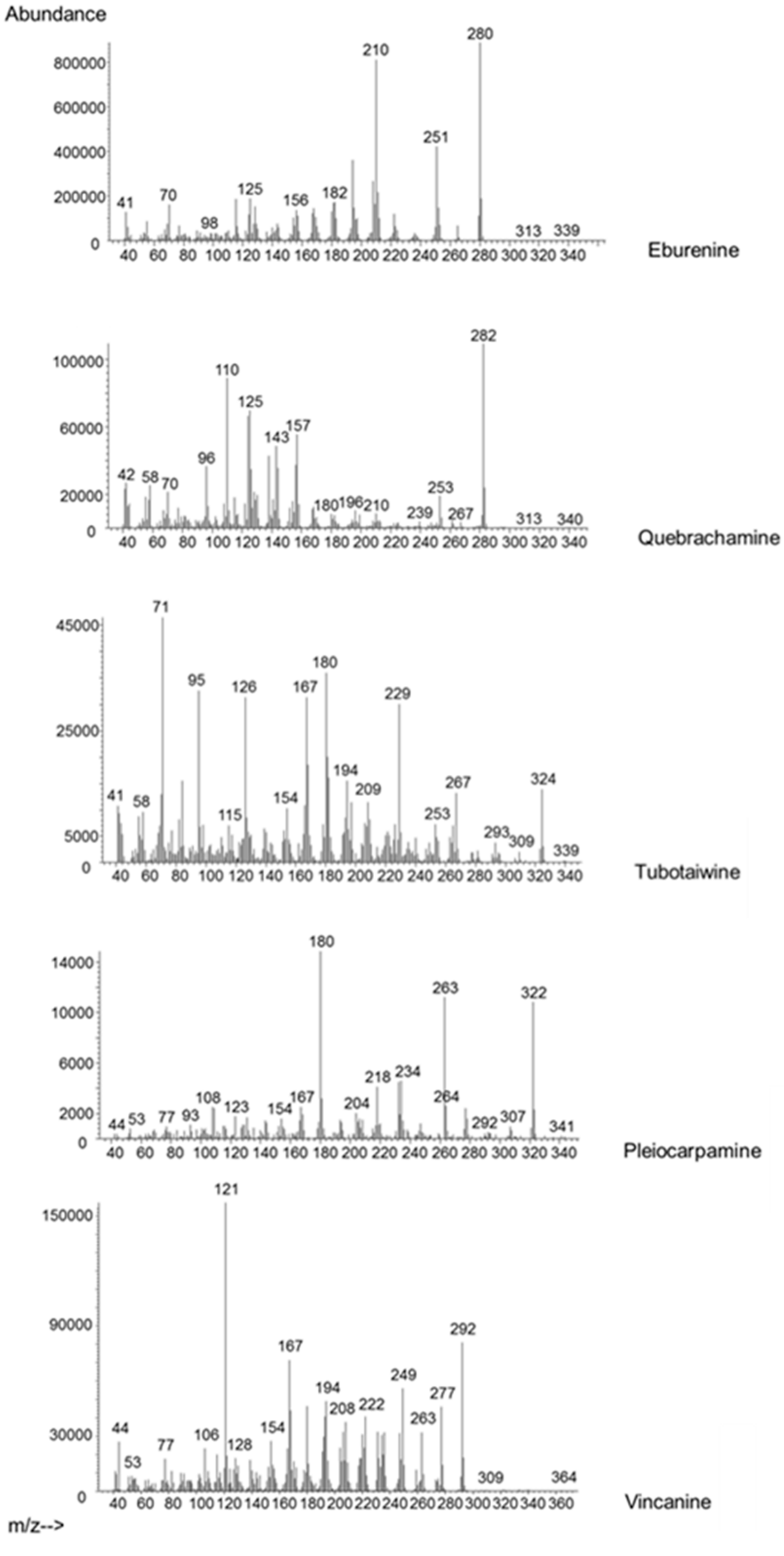

Abundance
210 280 251 41 70 125 156 182 98 313 339
Eburenine
282 110 125 143 157 96 42 58 70 180 196 210 239 253 267 313 340
Quebrachamine
71 180 95 126 167 229 194 209 41 58 115 154 253 267 324 293 309 339
Tubotaiwine
180 263 322 218 234 108 167 204 264 44 53 77 93 123 154 292 307 341
Pleiocarpamine
121 167 292 194 208 222 249 277 263 44 53 77 106 128 154 309 364
Vincanine
m/z-->

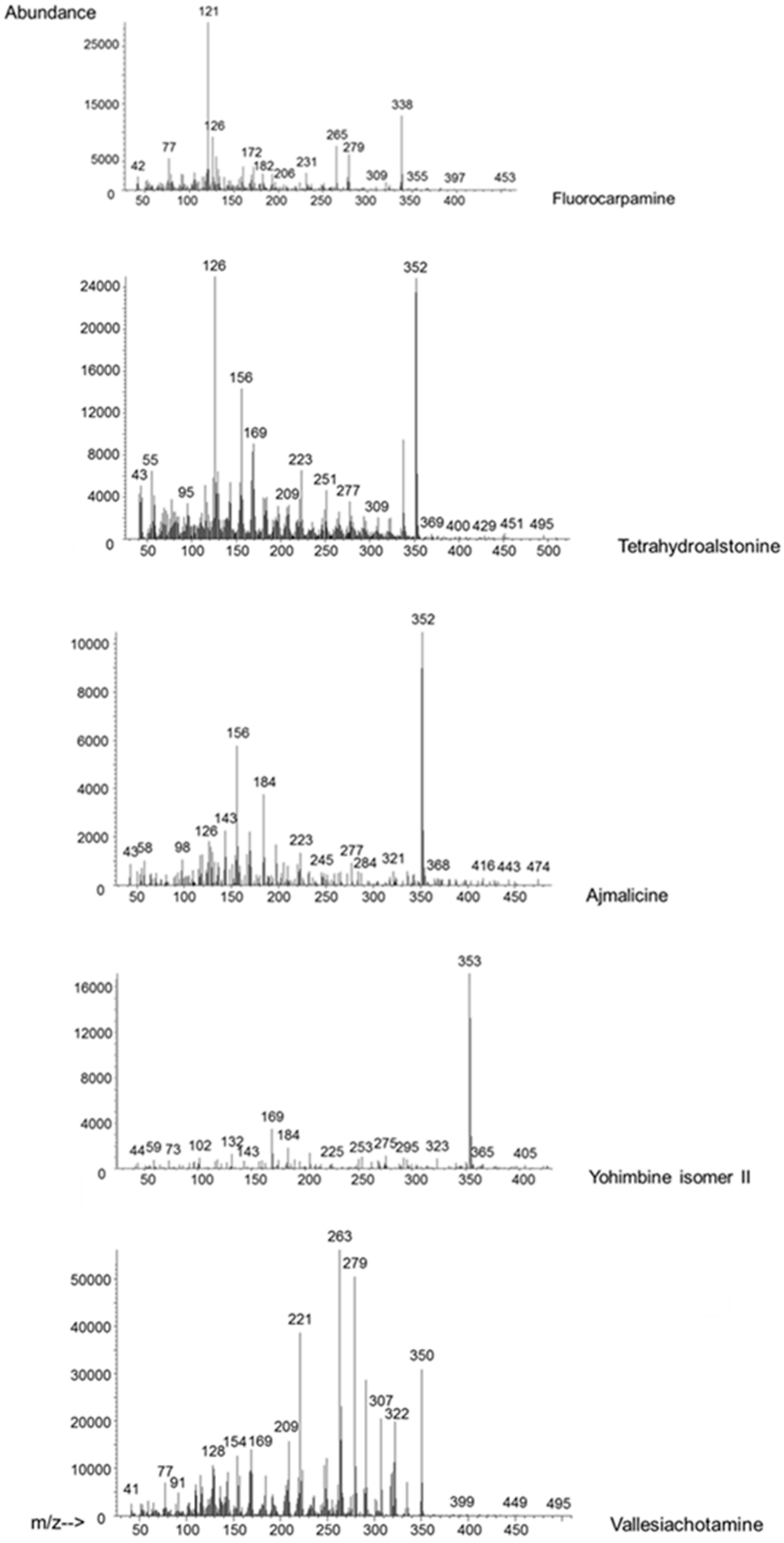

Figure 2. GC-MS spectra of the target alkaloids.
